# Exosomal hsa_circ_000200 as a potential biomarker and metastasis enhancer of gastric cancer via miR-4659a/b-3p/HBEGF axis

**DOI:** 10.1186/s12935-023-02976-w

**Published:** 2023-08-01

**Authors:** Xiao-juan Huang, Yan Wang, Hui-ting Wang, Zhao-feng Liang, Cheng Ji, Xiao-xi Li, Lei-lei Zhang, Run-bi Ji, Wen-rong Xu, Jian-hua Jin, Hui Qian

**Affiliations:** 1 Wujin Institute of Molecular Diagnostics and Precision Cancer Medicine of Jiangsu University, Wujin Hospital Affiliated with Jiangsu University, 2 Yong Ning North Road, Chang Zhou, 213017 Jiangsu China; 2 Jiangsu Key Laboratory of Medical Science and Laboratory Medicine, School of Medicine, Jiangsu University, 301 Xuefu Road, Zhenjiang, 212013 Jiangsu China; 3 Kunshan Hospital Affiliated with Jiangsu University, 91 Qianjin West Road, Kunshan, 215300 Jiangsu China; 4 The Laboratory Department, The Affiliated People’s Hospital of Jiangsu University, Zhenjiang, 212002 Jiangsu China

**Keywords:** Exosomes, hsa_circ_000200, Gastric cancer, Liquid biopsy marker, miR-4659a/b-3p, HBEGF

## Abstract

**Background:**

Exosome, a component of liquid biopsy, loaded protein, DNA, RNA and lipid gradually emerges as biomarker in tumors. However, exosomal circRNAs as biomarker and function mechanism in gastric cancer (GC) are not well understood.

**Methods:**

Differentially expressed circRNAs in GC and healthy people were screened by database. The identification of hsa_circ_000200 was verified by RNase R and sequencing, and the expression of hsa_circ_000200 was evaluated using qRT-PCR. The biological function of hsa_circ_000200 in GC was verified in vitro. Western blot, RIP, RNA fluorescence in situ hybridization, and double luciferase assay were utilized to explore the potential mechanism of hsa_circ_000200.

**Results:**

Hsa_circ_000200 up-regulated in GC tissue, serum and serum exosomes. Hsa_circ_000200 in serum exosomes showed better diagnostic ability than that of tissues and serum. Combined with clinicopathological parameters, its level was related to invasion depth, TNM staging, and distal metastasis. Functionally, knockdown of hsa_circ_000200 inhibited GC cells proliferation, migration and invasion in vitro, while its overexpression played the opposite role. Importantly, exosomes with up-regulated hsa_circ_000200 promoted the proliferation and migration of co-cultured GC cells. Mechanistically, hsa_circ_000200 acted as a “ceRNA” for miR-4659a/b-3p to increase HBEGF and TGF-β/Smad expression, then promoted the development of GC.

**Conclusions:**

Our findings suggest that hsa_circ_000200 promotes the progression of GC through hsa_circ_000200/miR-4659a/b-3p/HBEGF axis and affecting the expression of TGF-β/Smad. Serum exosomal hsa_circ_000200 may serve as a potential biomarker for GC.

**Supplementary Information:**

The online version contains supplementary material available at 10.1186/s12935-023-02976-w.

## Introduction

Gastric cancer (GC) is a common malignant tumor with high morbidity and mortality [1]. The histopathological examination has been considered to be the gold standard for diagnosing GC [2], but it is invasive, highly heterogeneous, and prone to complications [3]. Tumor markers, as early clinical diagnosis and prognostic indicators, are less invasive and readily accepted by GC patients. So far, carcinoembryonic antigen (CEA) and CA19-9 are routinely used serum tumor biomarkers in clinical practice, but their sensitivity and specificity are not high, and they still have some limitations in the diagnosis and therapy of GC [4, 5]. Therefore, it is crucial to explore a sensitive and specific non-invasive marker for GC.

Over the past decade, liquid biopsy has attracted extensive attention in the field of oncology diagnosis, efficacy and prognosis due to its low invasiveness. Exosome, one of contents of liquid biopsy, has gradually become the new star in cancer for its stable structure, high abundance and rich in bioactive substances. Exosomes are small extracellular vesicles (sEVs) with a diameter of 30–150 nm, composed of phospholipid bilayers and abundant bioactive substances including proteins, messenger RNA (mRNA), microRNA (miRNA), and circular RNA (circRNA) [6]. CircRNAs are stable and enriched in exosomes. They are covalently closed endogenous biomolecules with cell-specific and tissue-specific expression patterns in eukaryotes that can act as miRNA sponges, translate proteins, and regulate epigenetic modifications [7]. Accumulating evidence indicates that exosomal circRNAs are closely related to the occurrence and development of tumors, and are important regulators of tumors [8–11]. In addition, previous studies have demonstrated that abundant and stable circRNAs can be detected in human blood exosomes and can be used as tumor non-invasive liquid biopsy biomarkers [12, 13].

In this study, we identified hsa_circ_000200 associated with GC, explored its clinical significance, function and potential mechanism in GC progression. We found that hsa_circ_000200 (CircBase ID: hsa_circ_0000670) was significantly up-regulated in tissues, serum and serum exosomes of GC patients. It promoted the development of GC by sponging miR-4659a/b-3p and increasing the expression of heparin-binding epidermal growth factor-like growth factor (HBEGF) and TGF-β/Smad. Our findings suggest that serum exosomal hsa_circ_000200 may be a biomarker and proved new insights for liquid biopsy of GC.

## Materials and methods

### Clinical specimens

A total of 98 pairs of GC tissues and adjacent nononcologic tissues (5 cm away from the tumor edge) were obtained from patients undergoing surgical excision at Department of General Surgery, the Affiliated People’s Hospital of Jiangsu University from 2018 to 2020. Serum specimens from 54 GC patients and 54 healthy controls were also collected during this period. The clinicopathological features were analyzed, including 95 tissue samples and 50 serum samples. All of the serum and tissue samples were aliquoted and stored at − 80 °C. This study was approved by the Medical Ethics Committee of Jiangsu University (2012258, 2022264).

### Cell culture and transfection

The HGC-27, MKN-45, AGS cell lines were purchased from Runke Biotechnology (Guangzhou, China) and the MGC803 and HEK-293T were from Cell Bank of the Chinese Academy of Sciences (Shanghai, China). Normal gastric mucosa epithelial cell line GES-1 was purchased from Gefan Biological Technology (Shanghai, China). The MGC-803 and HEK-293T were cultured in high-glucose DMEM (Bioind, Israel). The HGC-27, MKN-45 and GES-1 cells were maintained in RPMI-1640 (Bioind, Israel) and the AGS cells were propagated in DMEM/F-12 Medium (Bioind, Israel). All cell lines were supplemented with 10% fetal bovine serum (FBS; Excell, China) in a humid environment with 5% CO_2_ at 37 °C. The plasmid of hsa_circ_000200 (OE) was synthesized by BersinBio (Guangzhou, China), and ASO targeting hsa_circ_000200 was synthesized by RiboBio (Guangzhou, China). The sequence of ASO was TGTGTTGAAGGGACTGTTTA. Furthermore, miR-4659a/b-3p mimics or inhibitors were designed and synthesized by GenePharma (Suzhou, China). The plasmids, ASO and miRNA mimics or inhibitors were transfected into cells with Lipofectamine 2000 (Invitrogen, USA). RNA collection and cell function experiments were performed 48 h after transfection. Protein collection was performed 72 h after transfection.

### Exosome isolation and identification

Exosomes from human serum (serum-ex) were isolated from ExoQuick exosome precipitation solution (SBI, USA). Exosomes of cell origin were collected by super-centrifugation. The supernatant of GC cells was centrifuged at 2000×*g* for 10 min to remove precipitated cells, followed by 30 min at 10,000×*g* to remove cell debris, and then centrifuged at 100,000×*g* for 2 h and repeated once. The precipitate was dissolved in sterile PBS after super-centrifugation to obtain exosomes. All exosomes were stored at − 80 °C. To identify extracted exosomes, transmission electron microscopy and nanoparticle tracking analysis (NTA) were used to determine exosome form size and western blot was applied to evaluate exosome protein markers (CD63, CD81, TSG101 and Calnexin).

### Exosome co-culture assay

Hsa_circ_000200 had high content in MKN-45 exosomes and low content in AGS exosomes, so we collected and extracted MKN-45 exosomes (with a concentration of 1.3 × 10^8^ particles/mL) for co-culture with AGS. We digested the AGS in logarithmic growth phase and seeded it in a 6-well plate at a density of 2 × 10^5^, then cultured at 5% CO_2_ at 37 °C for adhesion. Added PBS, 20 μL exosomes/well (MKN-45-ex-20) and 40 μL exosomes/well (MKN-45-ex-40) to the corresponding hole. Changed the medium every 24 h and continued to add exosomes of the corresponding concentration. After 48 h of culture, we harvested cells for functional experiments.

### RNA fluorescence in situ hybridization (FISH)

The specific probe of hsa_circ_000200 was labeled by Cy3 and synthesized by GenePharma and the signals were detected using FISH Kit (GenePharma, Suzhou, China). Grew exponential growth cells into 12-well plates and fixed when the cells were 40–50% fusion. Cells were permeabilized with 0.1% Triton X-100 and specifically hybridized with hsa_circ_000200, U6 and 18S overnight at 37 °C. Nuclei was stained with DAPI. Laser scanning confocal microscope (Nikon, Japan) was used for visualization and image acquisition.

### RNA preparation, RNase R, qRT-PCR and sequencing

Total RNA from serum and serum exosomes was separated using miRNeasy Serum/Plasma kit (Qiagen, Germany). Total RNA from cell lines and tissues was extracted and purified using TRIzol reagent (Gibco, USA). In the RNase R assay, 2 μg cell-derived total RNA was incubated at 37 °C for 15 min with 6 U/3 μL RNAse R (Epicentre, USA). These RNAs were transcribed into cDNA using HiScript IIIst Strand cDNA Synthesis Kit (Vazyme, Nanjing, China). Subsequently, qRT-PCR was executed on 7300 Plus (ABI, USA) with AceQ qpcr sybr green master mix (Vazyme, Nanjing, China). The qRT-PCR products were sent for sequencing (Sangon Biotech, Shanghai, China). β-actin was used as an internal control and the results for each sample were normalized to β-actin expression. The expression level was calculated by −∆Ct method and the relative expression level was presented by 2^−∆∆Ct^ method. Primer sequences of this study were shown in Table 1.

**Table 1:** Sequences of primers for qRT-PCR and miRNA related sequence

Name		Sequence
Hsa_circ_000200	Forward	GCATTCTTACTCTTAGGGTTCATAC
	Reverse	CCCTTCAACACAACACTGTCTTA
miR-4659a-3p	Forward	CGTCACCGTTTCTTCTTAGACA
miR-4659b-3p	Forward	CGACGTTTCTTCTTAGACATGG
miR-4659a/b-3p	Reverse	TATGGTTGTTCTGCTCTCTGTCTC
U6	Forward	CGCTTCGGCAGCATATAC
	Reverse	TTCACGAATTTGCGTGTCATC
β-actin	Forward	GACCTGTACGCCAACACAGT
	Reverse	CTCAGGAGGAGCAATGATCT
HBEGF	Forward	TCCTCTCGGTGCGGGACCAT
	Reverse	GTGCCGAGAGAACTGCAGCCAG

### Transwell assays and cell counting kit-8 (CCK8) assay

The migration experiment was similar to the invasion, but the difference was migration used normal transwell chamber (Corning, MA, USA) while the invasion used transwell chamber with matrix (BD Biosciences). 600 μL nutrient solution containing 10%FBS was added to the 24-well plate and placed the transwell chamber in the well for later use. After the cells were treated for 48 h, suspended them in non-FBS nutrient solution, and then seeded 100,000 cells into the transwell chamber at 200 μL. Incubated at 37 °C for 12–24 h, fixed with 4% paraformaldehyde and stained with crystal violet, the results were observed under a microscope. Cell proliferation assay was determined by CCK8. After the cells were transfected for 48 h, 1000 cells were suspended in 100 μL nutrient solution containing 10%FBS and seeded in 96-well plates, 10 μL CCK8 (Vazyme, Nanjing, China) was added to each well for 2 h. Then, using microplate reader (BioTEK, USA) to measure the absorbance at 450 nm. The above measurements were continued for 5 days.

### RNA–protein immunoprecipitation (RIP)

Using Magna RIP RNA-Binding Protein Immunoprecipitation Kit (Millipore, Billerica, USA) to perform RIP assay. According to the instruction manual of kit, cell lysate was incubated with magnetic beads conjugated to IgG antibodies and anti-Ago2 (Millipore, Billerica, USA). The system was rotated and incubated overnight at 4 °C. The purified RNA was detected by qRT-PCR.

### Western blot

Cells were lysed using RIPA buffer (Pierce, Shanghai, China) containing protease inhibitors to obtain total protein. Protein samples were separated by SDS-Polyacrylamide gel electrophoresis (SDS-PAGE) and then transferred to polyvinylidene fluoride (PVDF) membranes (Millipore, USA). The membrane was blocked in 5% skim milk for 2 h, then incubated with primary antibodies at 4℃ overnight. The primary antibodies were as follows: anti-β-actin (SAB, 21800-2); anti-Mmp9 (CST, 13667S); anti-E-cadherin (Bioworld, BS1098); anti-N-Cadherin (CST, 14215S); anti-Slug (CST, 9585P); anti-Twist (CST, 46702s); anti-Vimentin (Bioworld, BS1491); anti-PCNA (CST, 13110); anti-CyclinD1 (Bioworld, BS1741); anti-CD44 (Bioworld, BS6825); anti-Sox2 (CST, 23064S); anti-Oct4 (CST, 2750S); anti-Lin28 (Proteintech, 11724-1-AP); anti-Nanog (SAB, 21423); anti-CD63 (Proteintech, 25682-1-AP); anti-CD81 (Proteintech, 18250-1-AP); anti-TSG101 (Abcam, ab125011); anti- Calnexin (Abcam, ab22595); anti-HBEGF (SANTA CRUZ, sc-365182); anti-TGF-β (Bioworld, BS1361); anti-Smad2/3 (Wanleibio, WL01520); anti-P-Smad2/3 (Wanleibio, WL02305). Afterwards, the membrane was washed three times with Tris–HCl buffered salt solutions and Tween (TBST), then incubated at room temperature with the second antibody for 2 h. After washing by TBST three times, proteins on the membrane were visualized using enhanced chemiluminescence (ImageQuant LAS4000mini, GE, Japan).

### Dual luciferase reporter assay

A wild type or mut-hsa_circ_000200/HBEGF fragments were constructed and inserted into the pmirGLO’s downstream of luciferase reporter gene (GenePharma, Suzhou, China). We co-transfected with a mixture of luciferase reporter vectors into HEK-293T or GC cells to examine the hsa_circ_000200 and miR-4659a/b-3p, HBEGF and miR-4659a/b-3p binding abilities. After 48 h, the cells were collected and the luciferase activity of firefly and kidney were detected by Dual-Glo Luciferase Assay Kit (Vazyme, Nanjing, China).

### Statistical analysis

All data were statistically analyzed using SPSS 20.0 (IBM, Chicago, IL) and GraphPad Prism version 7.0 software (LaJolla, CA, USA). To statistically compare the significance of differences between two or more groups, we used the Student’s t-test and one-way ANOVA. ROC analysis was used to evaluate the diagnostic value of hsa_circ_000200 of GC. Kaplan–meier method and log-rank test curve of survival were used for survival analysis.* P* < 0.05 was considered statistically significant.

## Results

### Serum exosome-derived hsa_circ_000200 acts as a potential biomarker for GC diagnosis

**Fig. 1:**
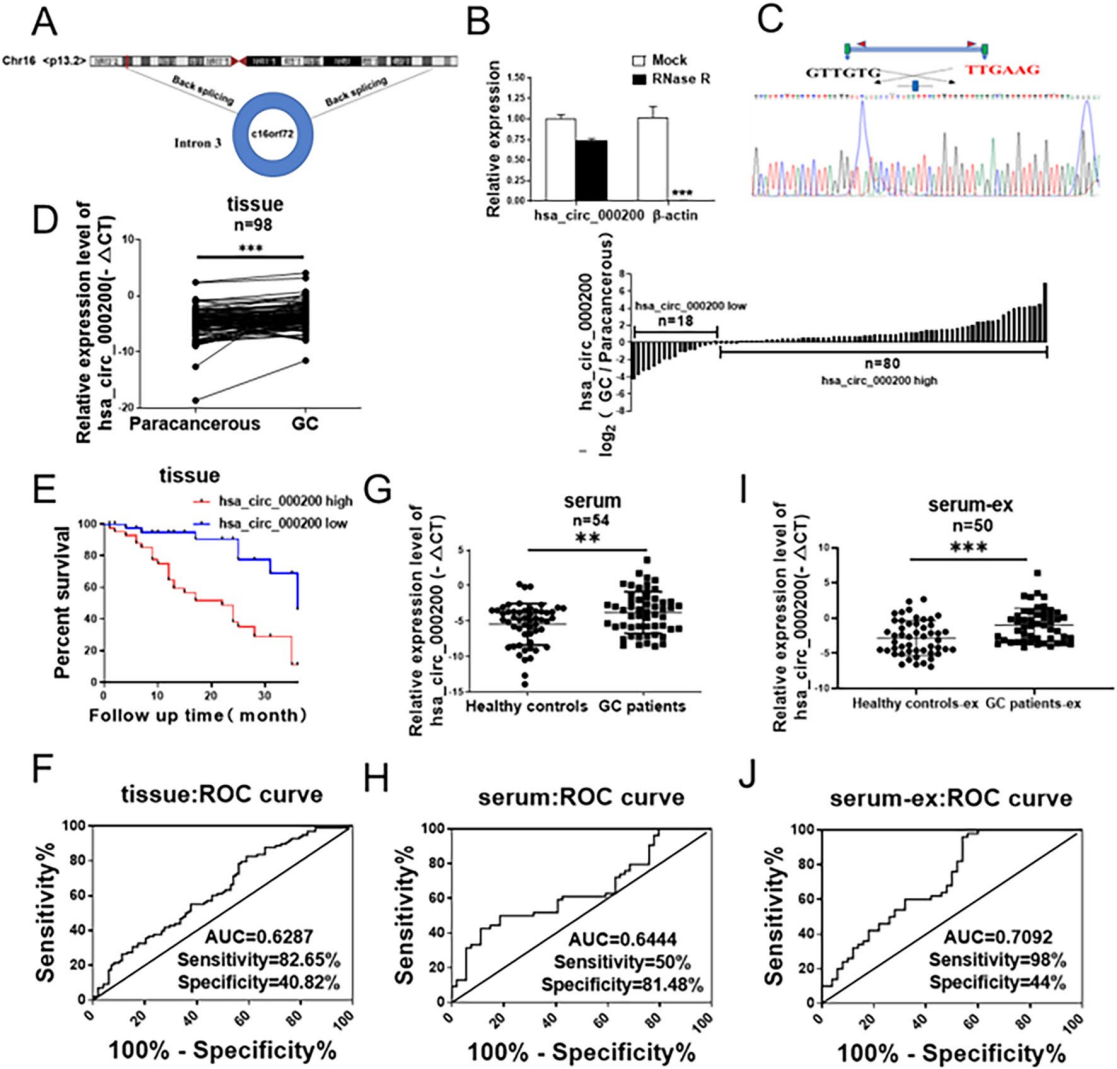
Serum exosomal hsa_circ_000200 acts as a potential biomarker for GC diagnosis. **A** Schematic diagram of the formation of hsa_circ_000200. **B** The level of hsa_circ_000200 under RNase R treatment compared with the level of β-actin. **C** The sequencing results of hsa_circ_000200. **D** The expression of hsa_circ_000200 in 98 paired of GC tissues and paracancer tissues. **E** Survival analysis of GC patients according to the expression of hsa_circ_000200 in tissues. **F** The area under the ROC curve of the GC tissues (Sensitivity = 82.65%, Specificity = 40.82%). **G** The expression of hsa_circ_000200 in 54 paired of GC patient serum. **H** The area under the ROC curve of the GC patient serum (Sensitivity = 50%, Specificity = 81.48%). **I** The expression of hsa_circ_000200 in 50 paired of GC patient serum-derived exosomes. **J** The area under the ROC curve of the GC patient serum-derived exosomes (Sensitivity = 98%, Specificity = 44%). *P < 0.05, **P < 0.01, ***P < 0.001

According to the circRNA microarray of Gene Expression Omnibus (GEO, https://www.ncbi.nlm.nih.gov/geo/) database (GSE83521 and GSE93541), we screened out the highly expressed circRNA-hsa_circ_000200. Hsa_circ_000200 was back spliced by intron 3 of ORF72 on chromosome 16 (Fig. 1A). Then we designed the specific primers for hsa_circ_000200. The dissolution curve of the primers was unimodal, and agarose gel electrophoresis proved that the length of the product was consistent with the designed theoretical length (Additional file 1: Fig. S1A, B). RNase R assay and sequencing verified its existence and primers were designed successfully (Fig. 1B, C). We found that hsa_circ_000200 was highly expressed in GC tissues (Fig. 1D), which was related to the shorter survival time of GC patients (Fig. 1E). Pearson correlation analysis showed that the expression of hsa_circ_000200 in tissues was correlated with invasion depth and TNM stage (Table 2). In addition, the particle size, electron microscope morphology and surface markers of extracted serum exosomes were shown in Additional file 1: Fig. S1C–E. Hsa_circ_000200 was elevated in serum and serum exosomes from GC patients (Fig. 1G, I). Its expression of serum exosomes was associated with distal metastasis, CA199 and CA125 (Table 3). The area under the ROC curve (AUC) of hsa_circ_000200 in tissues, serum and serum exosomes were 0.6287, 0.6444 and 0.7092 respectively, suggesting that it had better diagnostic efficiency for serum exosomes (Fig. 1F, H, J). Collectively, these results illustrate that hsa_circ_000200 is consistently highly expressed in tissues, serum and serum exosomes, being mainly related to the metastasis of GC, acting as the potential biomarker.

**Table 2:** Correlation between hsa_circ_000200 expression and clinical pathological characteristic in GC (n = 95)

Features	Number	High	Low	*P* value
Gender				
Male	68 (72%)	57 (72%)	11 (69%)	0.768
Female	27 (28%)	22 (28%)	5 (31%)	
Age (years)				
< 60	19 (20%)	15 (19%)	4 (25%)	0.732
≥ 60	76 (80%)	64 (81%)	12 (75%)	
Tumor size (cm)				
< 5	43 (45%)	33 (42%)	10 (63%)	0.171
≥ 5	52 (55%)	46 (58%)	6 (37%)	
Lymphatic metastasis				
N0	20 (21%)	15 (19%)	5 (31%)	0.316
N1–3	75 (79%)	64 (81%)	11 (69%)	
Distal metastasis				
M0	93 (98%)	77 (97%)	16 (100%)	0.988
M1	2 (2%)	2 (3%)	0 (0%)	
Invasion depth				
T1 and T2	5 (5%)	1 (1%)	4 (25%)	0.003**
T3 and T4	90 (95%)	78 (99%)	12 (75%)	
TNM stage				
I and II	20 (21%)	13 (16%)	7 (44%)	0.038*
III and IV	75 (79%)	66 (84%)	9 (56%)	

*P＜0.05, **P＜0.01

**Table 3:** Correlation between hsa_circ_000200 expression and clinical pathological characteristic in GC serum exosomes (n = 50)

Features	Number	High	Low	*P* value
Gender				
Male	39 (78%)	20 (77%)	19 (79%)	0.848
Female	11 (22%)	6 (23%)	5 (21%)	
Age (years)				
< 60	10 (20%)	5 (19%)	5 (21%)	0.887
≥ 60	40 (80%)	21 (81%)	19 (79%)	
Tumor size (cm)				
< 5	29 (58%)	12 (60%)	17 (57%)	0.815
≥ 5	21 (42%)	8 (40%)	13 (43%)	
Lymphatic metastasis				
N0	16 (32%)	9 (28%)	7 (39%)	0.434
N1–3	34 (68%)	23 (72%)	11 (61%)	
Distal metastasis				
M0	45 (90%)	20 (80%)	25 (100%)	0.018*
M1	5 (10%)	5 (20%)	0 (0%)	
Invasion depth				
T1 and T2	12 (24%)	4 (16%)	8 (32%)	0.185
T3 and T4	38 (76%)	21 (84%)	17 (68%)	
TNM stage				
I and II	19 (38%)	9 (30%)	10 (50%)	0.153
III and IV	31 (62%)	21 (70%)	10 (50%)	
CA199				
Negative	41 (82%)	18 (69%)	23 (96%)	0.014*
Positive	9 (18%)	8 (31%)	1 (4%)	
CA125				
Negative	42 (84%)	19 (73%)	23 (96%)	0.028*
Positive	8 (16%)	7 (27%)	1 (4%)	

*P＜0.05

### Hsa_circ_000200 promotes the progression of GC, especially in metastasis

**Fig. 2:**
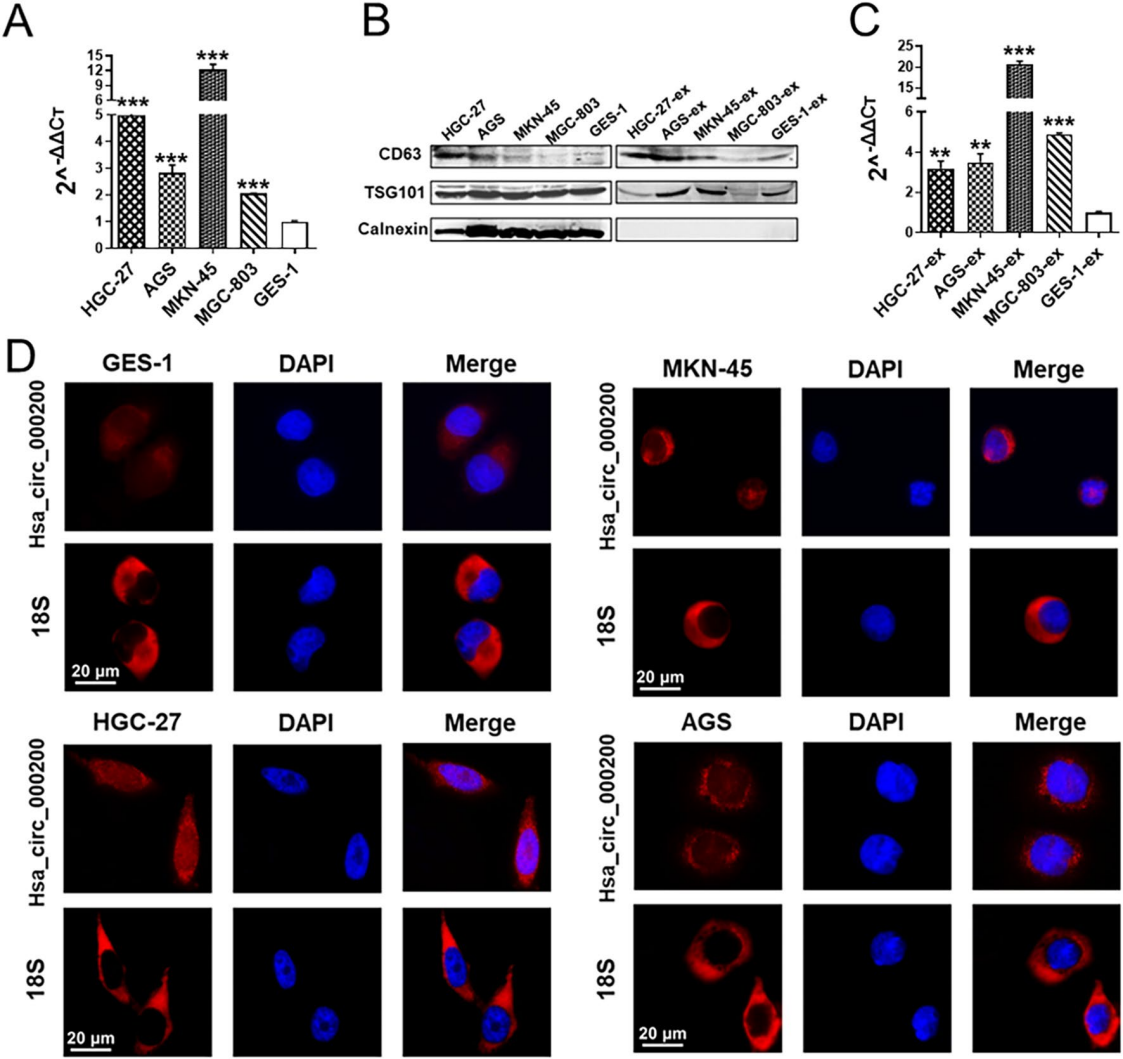
Expression and localization of hsa_circ_000200 in gastric cancer cell lines and its exosomes. **A** The expression of hsa_circ_000200 in GC cell lines. **B** Protein identification of cell and cell exosomes. **C** The expression of hsa_circ_000200 in exosomes of GC cell lines. **D** The localization of hsa_circ_000200 in GES-1, HGC-27, MKN-45 and AGS cells. *P < 0.05, **P < 0.01, ***P < 0.001

Next, we probed into the expression of hsa_circ_000200 in GC cells. The expression of hsa_circ_000200 in GC cells were higher than GES-1 cells, especially in MKN-45 and HGC-27 cells (Fig. 2A). We extracted and characterized exosomes derived from GC cells (Fig. 2B; Additional file 1: Fig. S1F, G). Hsa_circ_000200 was up-regulated in GC cell-derived exosomes, with the highest expression in MKN-45 cells (Fig. 2C). FISH assay showed that hsa_circ_000200 was principally located in the cytoplasm in MKN-45, AGS and GES-1 cells, while it was distributed in both the nucleus and the cytoplasm of HGC-27 cells (Fig. 2D). In GC and paired paracancer tissues, hsa_circ_000200 is roughly distributed in the cytoplasm (Additional file 1: Fig. S2).

**Fig. 3:**
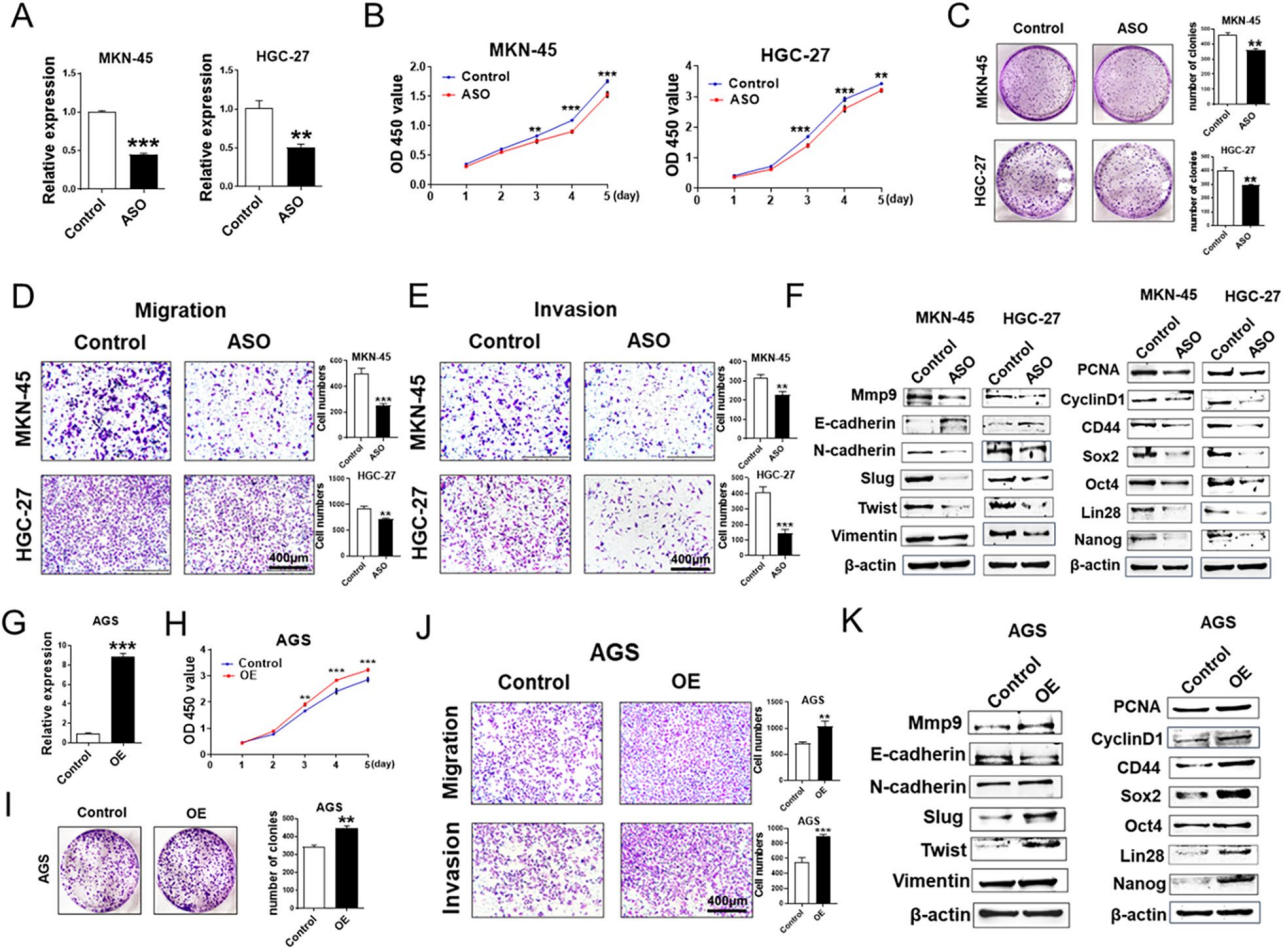
Hsa_circ_000200 promotes the progression of GC and maintains its stemness. **A** Hsa_circ_000200 knockdown efficiency is assessed by qRT-PCR. **B** Assessment of the proliferation in MKN-45 and HGC-27 cells transfected with control or hsa_circ_000200 ASO by CCK8 assay. **C** The number of cell clones after hsa_circ_000200 knockdown in MKN-45 and HGC-27 cells. **D** Hsa_circ_000200 knockdown inhibits the migration of MKN-45 and HGC-27 cells. **E** Hsa_circ_000200 knockdown inhibits the invasion of MKN-45 and HGC-27 cells. **F** The expression of proliferation, metastasis, and stemness-related proteins in GC cells after hsa_circ_000200 knockdown. **G** Hsa_circ_000200 overexpression efficiency is assessed by qRT-PCR. **H** Assessment of the proliferation in AGS cells transfected with control or hsa_circ_000200 plasmid by CCK8 assay. **I**. The number of cell clones after hsa_circ_000200 overexpression in AGS cells. **J** Hsa_circ_000200 overexpression promotes the migration and invasion of AGS cells. **K** The expression of proliferation, metastasis, and stemness-related proteins in GC cells after hsa_circ_000200 overexpression. *P < 0.05, **P < 0.01, ***P < 0.001

To further explore the role of hsa_circ_000200 in GC cells, we designed specific antisense oligonucleotide (ASO) to knockdown hsa_circ_000200 in MKN-45 and HGC-27 cells (Fig. 3A). The proliferation and clonal formation of MKN-45 and HGC-27 cells were decreased (Fig. 3B, C). The proportion of S phase cells was reduced after knockdown hsa_circ_000200 (Additional file 1: Fig. S3A). The ability of migration and invasion of GC cells was weakened after knockdown hsa_circ_000200 (Fig. 3D, E). Besides, western blot presented that the expression of metastasis related indicators such as Mmp9, N-cadherin, Slug, Twist and Vimentin were decreased, but E-cadherin was increased. The proliferation indicators PCNA, CyclinD1 and the stem cell markers CD44, Sox2, Oct4, Lin28, Nanog were weakened (Fig. 3F, Additional file 1: Fig. S5A, B). On the contrary, the proliferation, invasion and metastasis of AGS cells were enhanced after hsa_circ_000200 overexpression with plasmids (Fig. 3G–J; Additional file 1: Fig. S3B). Western blot results were opposite to hsa_circ_000200 knockdown (Fig. 3K, Additional file 1: Fig. S5C). Overall, hsa_circ_000200 knockdown inhibits the progress of GC, especially in metastasis, suggesting that it may play an essential role in the progression of GC as a metastasis-promoting factor.

### Exosomes transport hsa_circ_000200 to promote the progression of GC

**Fig. 4:**
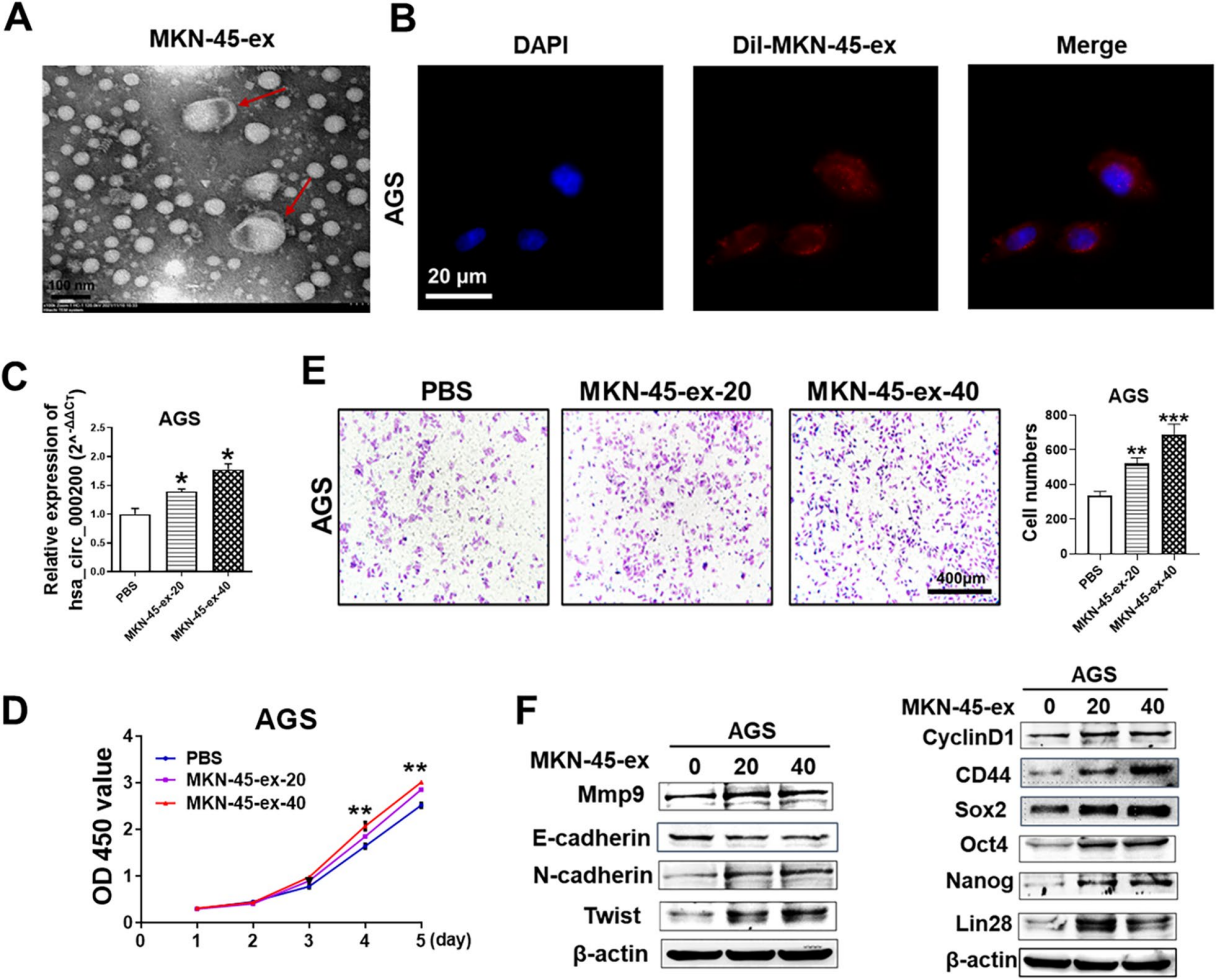
Exosomes transport hsa_circ_000200 to promote the progression of GC. **A** Morphology of exosomes derived from MKN-45 cells under transmission electron microscopy. **B** The results of AGS uptake of exosomes from MKN-45 cells. **C** Expression of hsa_circ_000200 in AGS cells after co-culture of exosomes. **D** Assessment of the proliferation in AGS cells treated with exosomes from MKN-45 cells by CCK8 assay. **E** Exosomes from MKN-45 cells promote the migration of AGS cells. **F** The expression of proliferation, metastasis, and stemness-related proteins in GC cells after co-culture of exosomes. *P < 0.05, **P < 0.01, ***P < 0.001

In order to further investigate whether hsa_circ_000200 can play a role in GC cells through exosome delivery. We first detected the expression of hsa_circ_000200 in exosomes of GC cells, and found that the expression of exosomes derived from MKN-45 cells was the highest, while AGS cells was less (Fig. 2C). Therefore, we collected and extracted exosomes from MKN-45 cells (Fig. 4A) and co-culture it with AGS cells. Fluorescent labeling results showed that AGS cells could ingest the added exosomes (Fig. 4B). The expression of hsa_circ_000200 in AGS cells appeared concentration-dependent of exosomes (Fig. 4C). After co-culture of exosomes, the cell cycle experiment showed that S phase cells were increased (Additional file 1: Fig. S3C), and the proliferation ability of AGS cells was enhanced (Fig. 4D). The migration of cells was enhanced (Fig. 4E). Western blot showed that the expression level of metastasis related indicators such as Twist, Mmp9 and N-cadherin were increased, while E-cadherin was decreased. The expression of stemness related indicators were increased, especially CD44, Nanog and Lin28 (Fig. 4F, Additional file 1: Fig. S5D). Taken together, hsa_circ_000200 enriched in exosomes derived from GC cells can be delivered to other surrounding cells to enhance proliferation and migration in GC, indicating that exosomes carrying hsa_circ_000200 act as a bridge of communication between GC cells.

### Hsa_circ_000200 acts as the sponge of miR-4659a/b-3p

**Fig. 5:**
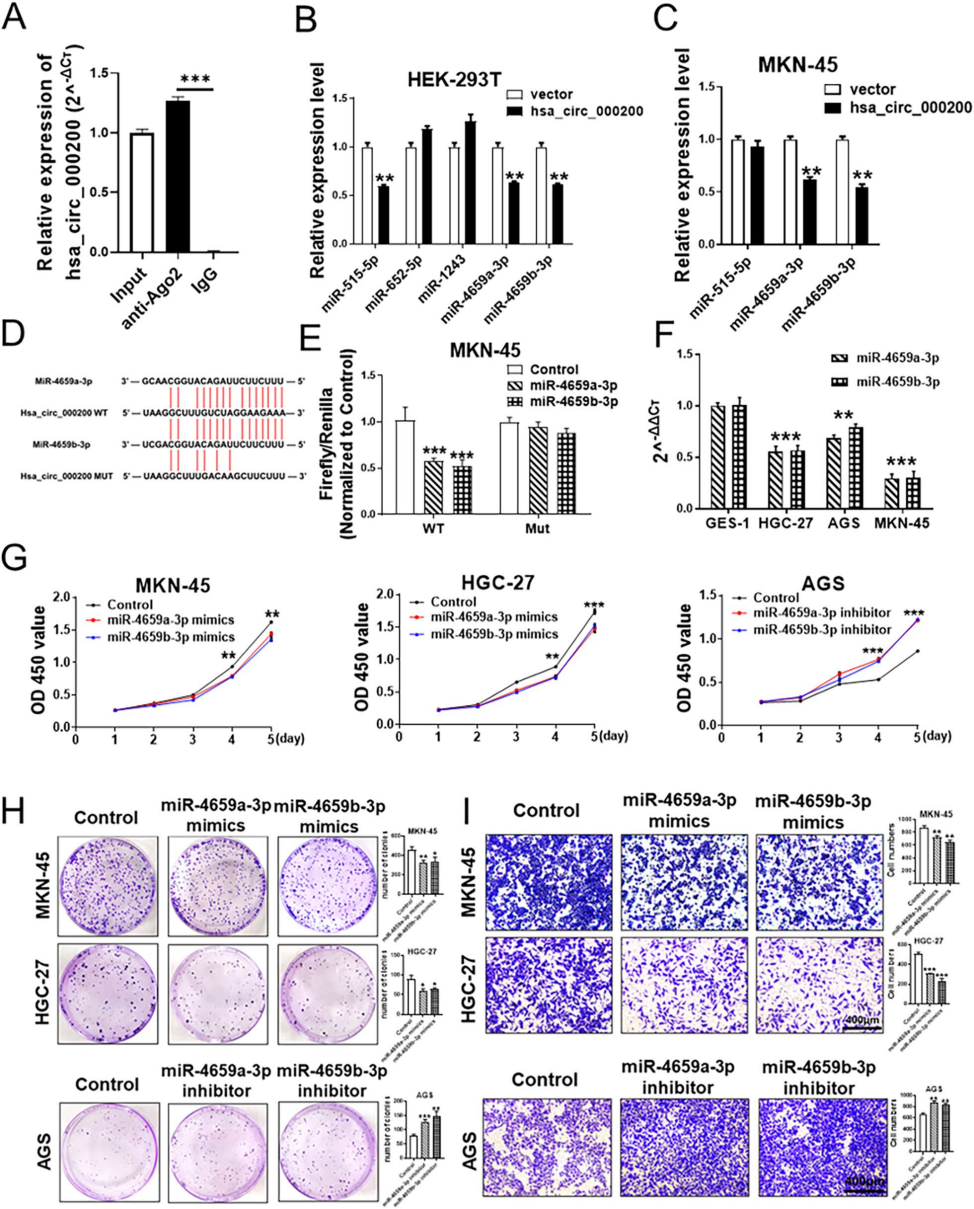
Hsa_circ_000200 acts as the sponge of miR-4659a/b-3p. **A** Level of hsa_circ_000200 detected by qRT-PCR after RIP for Ago2. **B** Validation of the levels of miRNAs that may bind to hsa_circ_000200 after overexpression of hsa_circ_000200 in HEK-293T cells. **C** Validation of the levels of miRNAs that may bind to hsa_circ_000200 after overexpression of hsa_circ_000200 in MKN-45 cells. **D** The potential binding site of miR-4659a/b-3p in hsa_circ_000200 is predicted by bioinformatic software. **E** The binding of hsa_circ_000200 and miR-4659a/b-3p is demonstrated by dual-luciferase reporter assay in MKN-45 cells. **F** Expression of miR-4659a/b-3p in GES-1, HGC-27, AGS and MKN-45. **G**. Assessment of the proliferation in MKN-45 and HGC-27 cells transfected with control or miR-4659a/b-3p mimics by CCK8 assay (**G** left and middle); assessment of the proliferation in AGS cells transfected with control or miR-4659a/b-3p inhibitor by CCK8 assay (**G** right). **H**. The number of cell clones in MKN-45 and HGC-27 after miR-4659a/b-3p mimics transfection (**H** above); the number of cell clones in AGS after miR-4659a/b-3p inhibitor transfection (**H** below). **I** The results of migration assays in MKN-45 and HGC-27 after miR-4659a/b-3p mimics transfection (**I** above); the results of migration assays in AGS after miR-4659a/b-3p inhibitor transfection (**I** below). *P < 0.05, **P < 0.01, ***P < 0.001

To explore the underlying function mechanism of hsa_circ_000200 in GC, FISH assay showed that hsa_circ_000200 was primarily located in the cytoplasm (Fig. 2D), suggesting that hsa_circ_000200 might play a role as miRNA sponge in GC. RIP assay proved that hsa_circ_000200 could combine with Ago2 (Fig. 5A). Bioinformatics prediction software (circbank, starbase and circinteractom) screened miRNAs (miR-515-5p, miR-652-5p, miR-1243, miR-4659a-3p and miR-4659b-3p) might bind to hsa_circ_000200. After overexpression of hsa_circ_000200, only the expression of miR-4659a/b-3p was down-regulated in HEK-293T and MKN-45 cells (Fig. 5B, C). Subsequently, we constructed a double luciferase reporter plasmid and their binding was experimentally confirmed (Fig. 5D, E). The expression of miR-4659a/b-3p was down-regulated in GC cell lines, and significantly in HGC-27 and MKN-45 (Fig. 5F). Next, we found that miR-4659a/b-3p acts as a tumor suppressor molecule in GC. After transfection with miR-4659a/b-3p mimics, the proliferation, clonal formation and migration of GC cells were reduced, while transfection with inhibitor had opposite results (Fig. 5G–I). These results indicated that miR-4659a/b-3p acts as a downstream of hsa_circ_000200 to inhibit the proliferation and migration of GC.

### Hsa_circ_000200 promotes metastasis and proliferation of GC via miR-4659a/b-3p/HBEGF axis

**Fig. 6:**
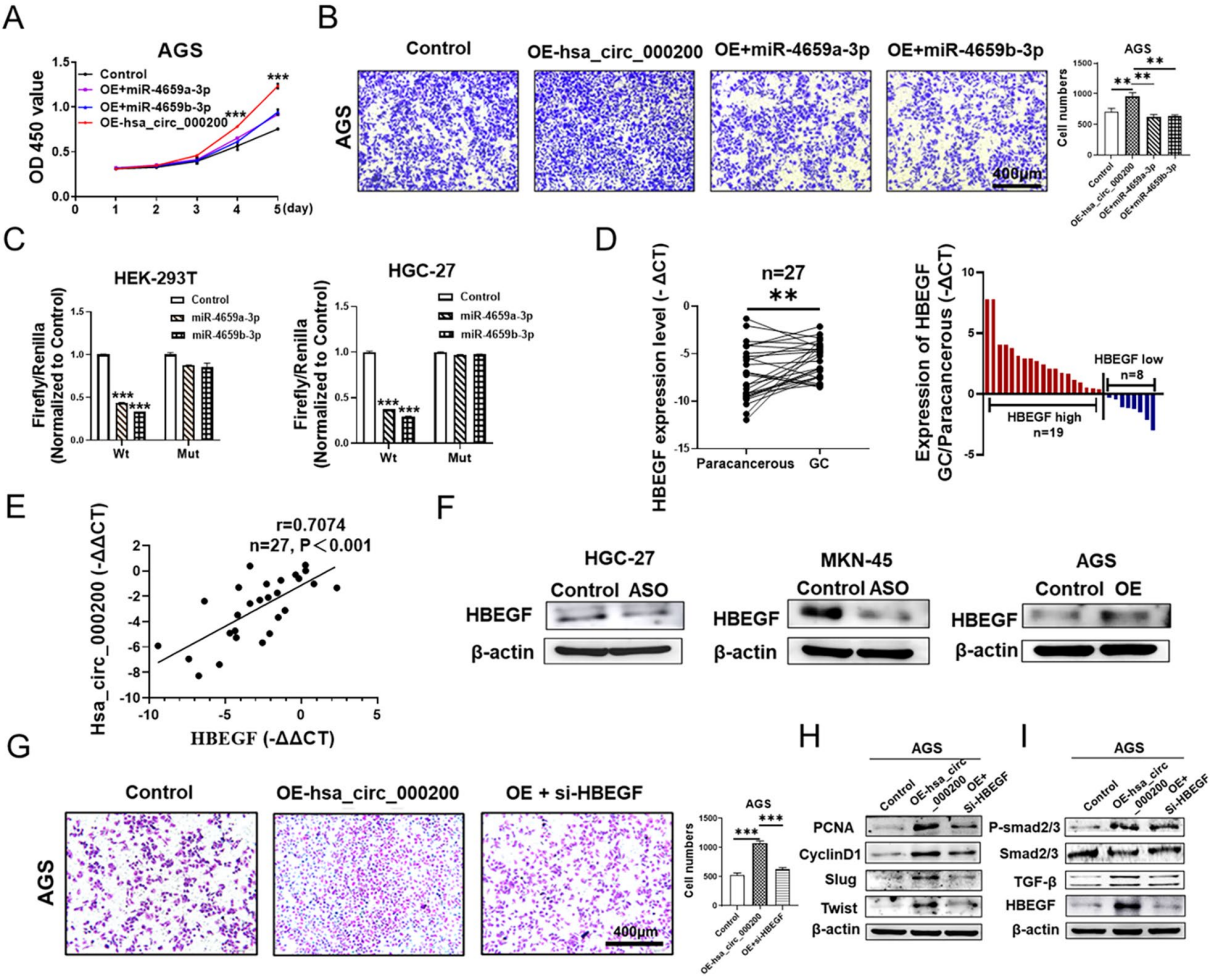
Hsa_circ_000200 promotes metastasis and proliferation of GC through regulating miR-4659a/b-3p/HBEGF axis. **A** Assessment of the proliferation in AGS cells transfected with hsa_circ_000200 plasmid and miR-4659a/b-3p mimics by CCK8 assay. **B** The results of migration assays in AGS after hsa_circ_000200 plasmid and miR-4659a/b-3p mimics transfection. **C** The binding of miR-4659a/b-3p and HBEGF is proved by dual-luciferase reporter assay in HEK-293T and HGC-27 cells. **D** The expression of HBEGF in 27 paired GC tissues and paracancer tissues. **E**. In the GC tissues, the level of hsa_circ_000200 and HBEGF were positively correlated by using Pearson correlation analysis. **F** The western blot results of HBEGF after hsa_circ_000200 ASO/plasmid transfection. **G** The results of migration assays in AGS after hsa_circ_000200 plasmid and si-HBEGF transfection. **H**, **I** The western blot results after hsa_circ_000200 plasmid and si-HBEGF transfection. *P < 0.05, **P < 0.01, ***P < 0.001

**Fig. 7:**
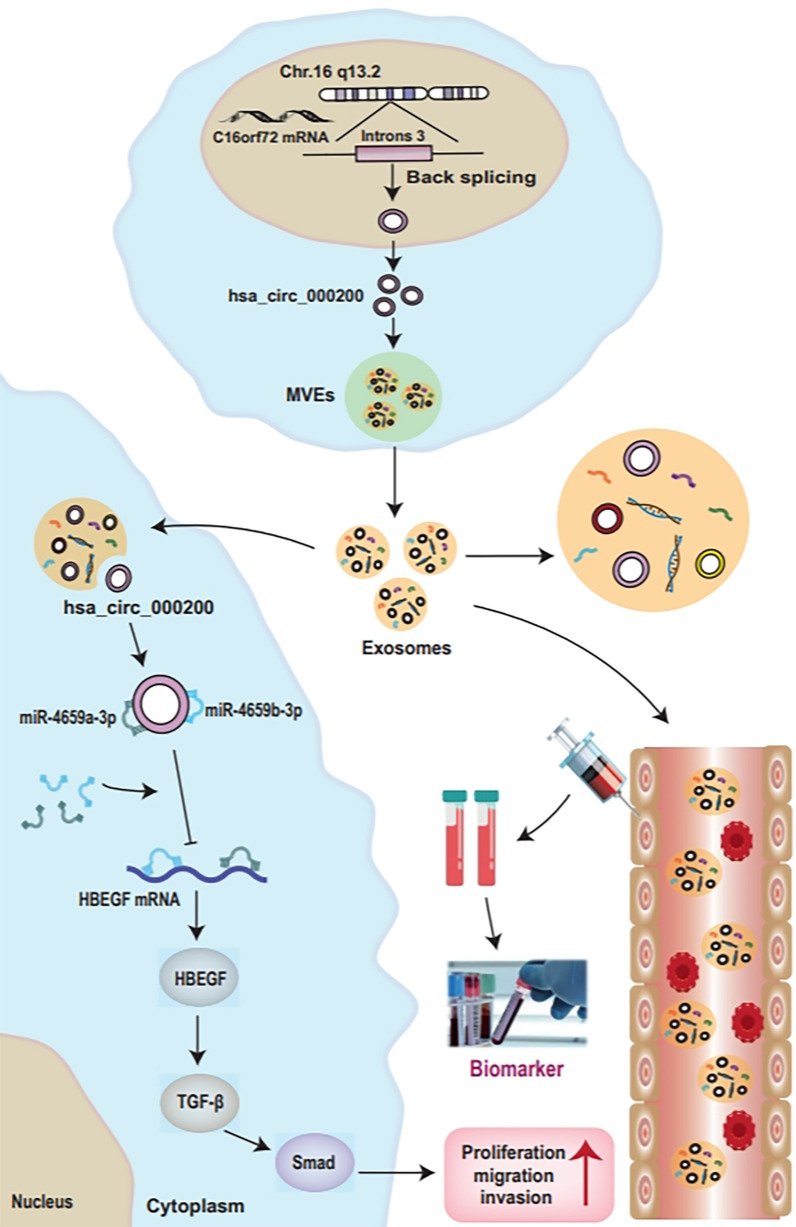
The mechanism diagram of the role of hsa_circ_000200 in GC

Furthermore, hsa_circ_000200 and miR-4659a/b-3p were simultaneously transfected in AGS cells. We found that miR-4659a/b-3p could reverse the promoting effect of hsa_circ_000200 on the proliferation and metastasis of GC (Fig. 6A, B). Next, the downstream target genes (HAS2, HBEGF, YES1, PTPN11, PRRG4) of miR-4659a/b-3p were predicted by bioinformatics software (TargetScan, miRTarBase and mirDB) and detected in GC cells using qRT-PCR. Among them, only HBEGF was consistent with the expression of hsa_circ_000200 and was opposite to miR-4659a/b-3p (Additional file 1: Fig. S4A–C). Dual luciferase reporting assay demonstrated that HBEGF could bind to miR-4659a/b-3p (Fig. 6C), the binding site was showed in Additional file 1: Fig. S4D. HBEGF was highly expressed in GC tissues (Fig. 6D). And there was a positive correlation between the expression level of HBEGF and hsa_circ_000200 (r = 0.7074, Fig. 6E). Further, western blot also confirmed that the expression of HBEGF increased after hsa_circ_000200 overexpression, while transfection with ASO had opposite results (Fig. 6F, Additional file 1: Fig. S5E, F). Transfection of si-HBEGF into AGS cells that overexpressing hsa_circ_000200 reversed the ability of promoting migration and proliferation (Fig. 6G; Additional file 1: Fig. S4E). Western blot showed that the expression of PCNA, CyclinD1, Slug and Twist were increased after overexpression of hsa_circ_000200 in AGS, while simultaneous knockdown of HBEGF reversed its promoting effect (Fig. 6H, Additional file 1: Fig. S5G). These findings suggest that HBEGF is a downstream target gene of miR-4659a/b-3p. By reviewing the literature, we found that the combined action of epidermal growth factor (EGF) and TGF-β signaling is a typical oncogenic cooperative and environmentally dependent mode [14]. Smad is a typical intracellular transcriptional effector of TGF-β receptor [15]. As a member of the EGF family, we suspect that HBEGF can also interact with TGF-β/Smad. Western blot results showed that after overexpression of hsa_circ_000200, the expression of HBEGF and TGF-β/Smad signaling pathway increased, and HBEGF knockdown reversed the promoting effect (Fig. 6I, Additional file 1: Fig. S5H). In summary, hsa_circ_000200 promotes metastasis and proliferation of GC by affecting the expression of TGF-β/Smad and miR-4659a/b-3p/HBEGF axis (Fig. 7).

## Discussion

GC is still a common malignant tumor that threatens human health at present. Its early symptoms are atypical, easy to be ignored and develop into advanced GC, resulting in poor treatment effect [16, 17]. In order to improve the early diagnosis rate and overcome the limitations of invasive tissue biopsy, liquid biopsy came into being. Liquid biopsy is an informative and minimally invasive tool with almost all tumor molecular characteristics, which can monitor tumor occurrence, metastasis, recurrence in real time, and evaluate treatment effect [18]. Liquid biopsy mainly isolates tumor-derived entities, such as circulating tumor DNA, circulating tumor cells, and tumor extracellular vesicles, then analyzes the genomic and proteomic data contained therein [19]. Saliva, blood, urine, pleural effusion, and cerebrospinal fluid can be used as liquid biopsy specimens. Because of the greatest contact between blood and tumor, most liquid biopsies utilize blood as the sample source [20]. Consequently, the search for blood-derived liquid biopsy markers is anticipated to enhance the early diagnosis rate of GC.

Accumulating evidence has shown that circRNAs are closely related to the occurrence and development of GC [21–24]. However, there are numerous types of circRNAs, their functions and mechanisms in GC are still unknown. In this study, we found hsa_circ_000200 was significantly up-regulated in GC cells and tissues, which consistent with previous literature reports [25]. In addition, since exosomes are an essential part of liquid biopsy [26], we detected hsa_circ_000200 in serum exosomes and found it was also significantly highly expressed in serum exosomes of GC patients and significantly correlated with the metastasis of GC, suggesting that exosomal hsa_circ_000200 may have potential as a diagnostic biomarker for GC.

The occurrence and development of tumors are inseparable from the proliferation, metastasis and intercellular communication. Here, we have confirmed that hsa_circ_000200 acts as an oncogene-like molecule and correlates with invasion depth, TNM stage and metastasis, indicating it may be an important molecule involved in GC progression. Exosomes are a class of small vesicles carrying a variety of biological information, which can participate in the information communication between tumor cells. CircRNAs can be enriched in exosomes [12]. We found hsa_circ_000200 was significantly increased in the serum exosomes of GC patients. It enriched in exosomes and transferred to the surrounding cells to promote cell proliferation and metastasis. In addition, we speculated exosomal hsa_circ_000200 can also be secreted into the blood, which makes hsa_circ_000200 highly expressed in the serum exosome of GC patients, thus distinguishing them from healthy people.

The mechanisms of circRNAs are diversified, with competitive endogenous RNAs (ceRNAs) that act as miRNAs being one of the crucial mechanisms. Increasing studies have shown that circRNAs can regulate the expression of tumor suppressor genes or oncogenes through the circRNA-miRNA-mRNA axis. For example, circ-ITCH can suppress the metastasis of GC cells by regulating the miR-199a-5p/Klotho axis [27]. CircRELL1 acts as a miR-637 sponge and inhibits the progression of GC by regulating autophagy activation [28]. Hsa_circ_0110389 acts as a sponge for miR-127-5p and miR-136-5p to up-regulate SORT1, which in turn promoted GC progression [29]. In this study, we firstly found that hsa_circ_000200 acts as a ceRNA for miR-4659a/b-3p and competitively bind miR-4659a/b-3p with HBEGF, thereby inhibiting the activity of miR-4659a/b-3p and enhancing the expression of HBEGF, TGF-β and P-smad, which in turn played a role in promoting GC progression. The role of miR-4659a/b-3p in GC has not been reported in the reference. Our study demonstrated that miR-4659a/b-3p can inhibit the proliferation and metastasis of GC cells and act as a bridge between hsa_circ_000200 and HBEGF for the first time. Studies have shown that Helicobacter pylori infection promotes the expression of HBEGF and induces the occurrence of GC [30]. HBEGF is highly expressed in GC tissues and can promote the metastasis of GC cells [31]. This is consistent with our results. We found that HBEGF is positively correlated with hsa_circ_000200 in GC tissues, suggesting that HBEGF may played a role as a tumor promoter. We also found that hsa_circ_000200 may affect the TGF-β/Smad signaling pathway through the miR-4659a/b-3p/HBEGF axis. However, whether the miR-4659a/b-3p/HBEGF axis can affect another signaling pathway remains to be explored. In addition, since hsa_circ_000200 was significantly enriched in exosomes, we speculate exosomal hsa_circ_000200 could also participate in this axis to promote gastric cancer metastasis.

Although we found exosomal hsa_circ_000200 was involved in the progression of GC and preliminarily explored its mechanism, there are still several limitations in this study. Firstly, hsa_circ_000200 was derived from database screening, but it is better to obtain differentially expressed circRNAs profiles by RNA-seq of serum exosomes from GC patients and healthy individuals. Secondly, the purpose of this study is to discover biomarkers for the diagnosis of GC, but the tumor heterogeneity is very strong, so the number of clinical samples needs to be further expanded. In addition, multiple circRNAs can be combined for diagnosis to improve the diagnostic efficiency.

## Conclusions

We have demonstrated hsa_circ_000200 is significantly highly expressed in GC patient tissues and serum, especially in serum exosomes, and is closely related to metastasis. Mechanistically, the occurrence and development of GC is promoted through hsa_circ_000200/miR-4659a/b-3p/HBEGF axis and affecting the expression of TGF-β/Smad. Therefore, our findings indicate that exosomal hsa_circ_000200 is considered to be an important communication molecule for GC progression and a potential biomarker for GC diagnosis.

## Supplementary Information

**Additional file 1: Figure S1.** A. The dissolution curve for hsa_circ_000200. B. The results of agarose gel electrophoresis. C. Nanoparticle Tracking Analysis (NTA) detected the particle size of serum exosomes in healthy controls and GC patients. D. The morphology of serum exosomes in healthy controls and GC patients under transmission electron microscope. E. Protein identification of serum exosomes in healthy controls and GC patients. F. NTA detected the particle size of exosomes from GES-1 cells. G. NTA detected the particle size of exosomes from MKN-45 cells. **Figure S2.** A/B/C. The localization of hsa_circ_000200 in GC and paired paracancer tissues. D. The localization of 18S in GC and paired paracancer tissues. **Figure S3.** A. The results of cell cycle experiments after hsa_circ_000200 knockdown in MKN-45 and HGC-27 cells. B. The results of cell cycle experiments after hsa_circ_000200 overexpression in AGS cells. C. The results of cell cycle experiments after co-culture of exosomes. **Figure S4.** A. Validation of the levels of mRNA that may bind to miR-4659a/b-3p after miR-4659a/b-3p mimics transfection in HGC-27 cells. B. Validation of the levels of HBEGF and PRRG4 after hsa_circ_000200 knockdown in HGC-27 cells. C. Validation of the levels of HBEGF and PRRG4 after hsa_circ_000200 overexpression in AGS cells. D. The potential binding site of HBEGF in miR-4659a/b-3p was predicted by bioinformatic software. E. Assessment of the proliferation in AGS cells transfected with hsa_circ_000200 plasmid and si-HBEGF by CCK8 assay. **Figure S5.** A-H. Relative levels of protein. *P < 0.05; **P < 0.01; ***P < 0.001.

## Data Availability

All data needed to evaluate the conclusions of the paper are contained in the paper and/or supplementary materials.

## Abbreviations

GC: Gastric cancer
circRNA: Circular RNA
sEVs: Small extracellular vesicles
CEA: Carcinoembryonic antigen
AUC: The area under the ROC curve
qRT-PCR: Quantitative reverse transcription polymerase chain reaction
ceRNA: Competitive endogenous RNA
FISH: Fluorescence in situ hybridization
RIP: RNA–protein immunoprecipitation
AGO2: Argonaute-2
HBEGF: Heparin-binding epidermal growth factor-like growth factor
ASO: Antisense oligonucleotide
CCK-8: Cell counting kit-8
TBST: Tris–HCl buffered salt solutions and Tween

## References

[1] Sung H, Ferlay J, Siegel RL, Laversanne M, Soerjomataram I, Jemal A, Bray F (2021). Global cancer statistics 2020: GLOBOCAN estimates of incidence and mortality worldwide for 36 cancers in 185 countries. CA Cancer J Clin.

[2] Wang FH, Zhang XT, Li YF, Tang L, Qu XJ, Ying JE, Zhang J, Sun LY, Lin RB, Qiu H, Wang C, Qiu MZ, Cai MY, Wu Q, Liu H, Guan WL, Zhou AP, Zhang YJ, Liu TS, Bi F, Yuan XL, Rao SX, Xin Y, Sheng WQ, Xu HM, Li GX, Ji JF, Zhou ZW, Liang H, Zhang YQ, Jin J, Shen L, Li J, Xu RH (2021). The Chinese Society of Clinical Oncology (CSCO): clinical guidelines for the diagnosis and treatment of gastric cancer, 2021. Cancer Commun (Lond).

[3] Levy I, Gralnek IM (2016). Complications of diagnostic colonoscopy, upper endoscopy, and enteroscopy. Best Pract Res Clin Gastroenterol.

[4] Matsuoka T, Yashiro M (2018). Biomarkers of gastric cancer: current topics and future perspective. World J Gastroenterol.

[5] Feng F, Tian Y, Xu G, Liu Z, Liu S, Zheng G, Guo M, Lian X, Fan D, Zhang H (2017). Diagnostic and prognostic value of CEA, CA19-9, AFP and CA125 for early gastric cancer. BMC Cancer.

[6] Kalluri R, Lebleu VS (2020). The biology, function, and biomedical applications of exosomes. Science.

[7] Kristensen LS, Andersen MS, Stagsted L, Ebbesen KK, Kjems J (2019). The biogenesis, biology and characterization of circular RNAs. Nat Rev Genet.

[8] Gao F, Xu Q, Tang Z, Zhang N, Huang Y, Li Z, Dai Y, Yu Q, Zhu J (2022). Exosomes derived from myeloid-derived suppressor cells facilitate castration-resistant prostate cancer progression via S100A9/circMID1/miR-506-3p/MID1. J Transl Med.

[9] Zhang PF, Gao C, Huang XY, Lu JC, Guo XJ, Shi GM, Cai JB, Ke AW (2020). Cancer cell-derived exosomal circUHRF1 induces natural killer cell exhaustion and may cause resistance to anti-PD1 therapy in hepatocellular carcinoma. Mol Cancer.

[10] Jiang Y, Zhao J, Xu J, Zhang H, Zhou J, Li H, Zhang G, Xu K, Jing Z (2022). Glioblastoma-associated microglia-derived exosomal circKIF18A promotes angiogenesis by targeting FOXC2. Oncogene.

[11] Tang B, Zhang Q, Liu K, Huang Y (2022). Exosomal circRNA FNDC3B promotes the progression of esophageal squamous cell carcinoma by sponging miR-490-5p and regulating thioredoxin reductase 1 expression. Bioengineered.

[12] Li Y, Zheng Q, Bao C, Li S, Guo W, Zhao J, Chen D, Gu J, He X, Huang S (2015). Circular RNA is enriched and stable in exosomes: a promising biomarker for cancer diagnosis. Cell Res.

[13] Roy S, Kanda M, Nomura S, Zhu Z, Toiyama Y, Taketomi A, Goldenring J, Baba H, Kodera Y, Goel A (2022). Diagnostic efficacy of circular RNAs as noninvasive, liquid biopsy biomarkers for early detection of gastric cancer. Mol Cancer.

[14] Sundqvist A, Vasilaki E, Voytyuk O, Bai Y, Morikawa M, Moustakas A, Miyazono K, Heldin CH, Ten Dijke P, van Dam H (2020). TGFβ and EGF signaling orchestrates the AP-1- and p63 transcriptional regulation of breast cancer invasiveness. Oncogene.

[15] Massagué J (2012). TGFβ signalling in context. Nat Rev Mol Cell Biol.

[16] Smyth ECNM, Grabsch HI, van Grieken NC, Lordick F (2020). Gastric cancer. Lancet.

[17] van Amelsfoort RM, Walraven I, Kieffer J, Jansen EPM, Cats A, van Grieken NCT, Meershoek-Klein Kranenbarg E, Putter H, van Sandick JW, Sikorska K, van de Velde CJH, Aaronson NK, Verheij M (2022). Quality of life is associated with survival in patients with gastric cancer: results from the randomized CRITICS trial. J Natl Compr Cancer Netw.

[18] Markou A, Tzanikou E, Lianidou E (2022). The potential of liquid biopsy in the management of cancer patients. Semin Cancer Biol.

[19] Lone SN, Nisar S, Masoodi T, Singh M, Rizwan A, Hashem S, El-Rifai W, Bedognetti D, Batra SK, Haris M, Bhat AA, Macha MA (2022). Liquid biopsy: a step closer to transform diagnosis, prognosis and future of cancer treatments. Mol Cancer.

[20] Pantel K, Alix-Panabieres C (2010). Circulating tumour cells in cancer patients: challenges and perspectives. Trends Mol Med.

[21] Zhang Y, Jiang J, Zhang J, Shen H, Wang M, Guo Z, Zang X, Shi H, Gao J, Cai H, Fang X, Qian H, Xu W, Zhang X (2021). CircDIDO1 inhibits gastric cancer progression by encoding a novel DIDO1-529aa protein and regulating PRDX2 protein stability. Mol Cancer.

[22] Zhang Y, Wang M, Zang X, Mao Z, Chen Y, Mao F, Qian H, Xu W, Zhang X (2020). CircHN1 affects cell proliferation and migration in gastric cancer. J Clin Lab Anal.

[23] Qiu S, Li B, Xia Y, Xuan Z, Li Z, Xie L, Gu C, Lv J, Lu C, Jiang T, Fang L, Xu P, Yang J, Li Y, Chen Z, Zhang L, Wang L, Zhang D, Xu H, Wang W, Xu Z (2022). CircTHBS1 drives gastric cancer progression by increasing INHBA mRNA expression and stability in a ceRNA- and RBP-dependent manner. Cell Death Dis.

[24] Li R, Jiang J, Shi H, Qian H, Zhang X, Xu W (2020). CircRNA: a rising star in gastric cancer. Cell Mol Life Sci.

[25] Liu P, Cai S, Li N (2020). Circular RNA-hsa-circ-0000670 promotes gastric cancer progression through the microRNA-384/SIX4 axis. Exp Cell Res.

[26] Vaidyanathan R, Soon RH, Zhang P, Jiang K, Lim CT (2018). Cancer diagnosis: from tumor to liquid biopsy and beyond. Lab Chip.

[27] Wang Y, Wang H, Zheng R, Wu P, Sun Z, Chen J, Zhang L, Zhang C, Qian H, Jiang J, Xu W (2021). Circular RNA ITCH suppresses metastasis of gastric cancer via regulating miR-199a-5p/Klotho axis. Cell Cycle.

[28] Sang H, Zhang W, Peng L, Wei S, Zhu X, Huang K, Yang J, Chen M, Dang Y, Zhang G (2022). Exosomal circRELL1 serves as a miR-637 sponge to modulate gastric cancer progression via regulating autophagy activation. Cell Death Dis.

[29] Liang M, Yao W, Shi B, Zhu X, Cai R, Yu Z, Guo W, Wang H, Dong Z, Lin M, Zhou X, Zheng Y (2021). Circular RNA hsa_circ_0110389 promotes gastric cancer progression through upregulating SORT1 via sponging miR-127-5p and miR-136-5p. Cell Death Dis.

[30] Baj J, Korona-Głowniak I, Forma A, Maani A, Sitarz E, Rahnama-Hezavah M, Radzikowska E, Portincasa P (2020). Mechanisms of the epithelial–mesenchymal transition and tumor microenvironment in helicobacter pylori-induced gastric cancer. Cells.

[31] Murayama Y, Miyagawa J, Shinomura Y, Kanayama S, Isozaki K, Yamamori K, Mizuno H, Ishiguro S, Kiyohara T, Miyazaki Y, Taniguchi N, Higashiyama S, Matsuzawa Y (2002). Significance of the association between heparin-binding epidermal growth factor-like growth factor and CD9 in human gastric cancer. Int J Cancer.

