# Supplementary material for: Exosomal hsa_circ_000200 as a potential biomarker and metastasis enhancer of gastric cancer via miR-4659a/b-3p/HBEGF axis
Source: Cancer Cell Int. 2023 Aug 1;23:151. doi: 10.1186/s12935-023-02976-w (PMC10391853; doi:10.1186/s12935-023-02976-w)
Supplement: Supplementary file 1 — Additional file 1: Figure S1. A. The dissolution curve for hsa_circ_000200. B. The results of agarose gel electrophoresis. C. Nanoparticle Tracking Analysis (NTA) detected the particle size of serum exosomes in healthy controls and GC patients. D. The morphology of serum exosomes in healthy controls and GC patients under transmission electron microscope. E. Protein identification of serum exosomes in healthy controls and GC patients. F. NTA detected the particle size of exosomes from GES-1 cells. G. NTA detected the particle size of exosomes from MKN-45 cells. Figure S2. A/B/C. The localization of hsa_circ_000200 in GC and paired paracancer tissues. D. The localization of 18S in GC and paired paracancer tissues. Figure S3. A. The results of cell cycle experiments after hsa_circ_000200 knockdown in MKN-45 and HGC-27 cells. B. The results of cell cycle experiments after hsa_circ_000200 overexpression in AGS cells. C. The results of cell cycle experiments after co-culture of exosomes. Figure S4. A. Validation of the levels of mRNA that may bind to miR-4659a/b-3p after miR-4659a/b-3p mimics transfection in HGC-27 cells. B. Validation of the levels of HBEGF and PRRG4 after hsa_circ_000200 knockdown in HGC-27 cells. C. Validation of the levels of HBEGF and PRRG4 after hsa_circ_000200 overexpression in AGS cells. D. The potential binding site of HBEGF in miR-4659a/b-3p was predicted by bioinformatic software. E. Assessment of the proliferation in AGS cells transfected with hsa_circ_000200 plasmid and si-HBEGF by CCK8 assay. Figure S5. A-H. Relative levels of protein. *P < 0.05; **P < 0.01; ***P < 0.001. [file 12935_2023_2976_MOESM1_ESM.docx]

**Additional file 1**

**Figure S1**


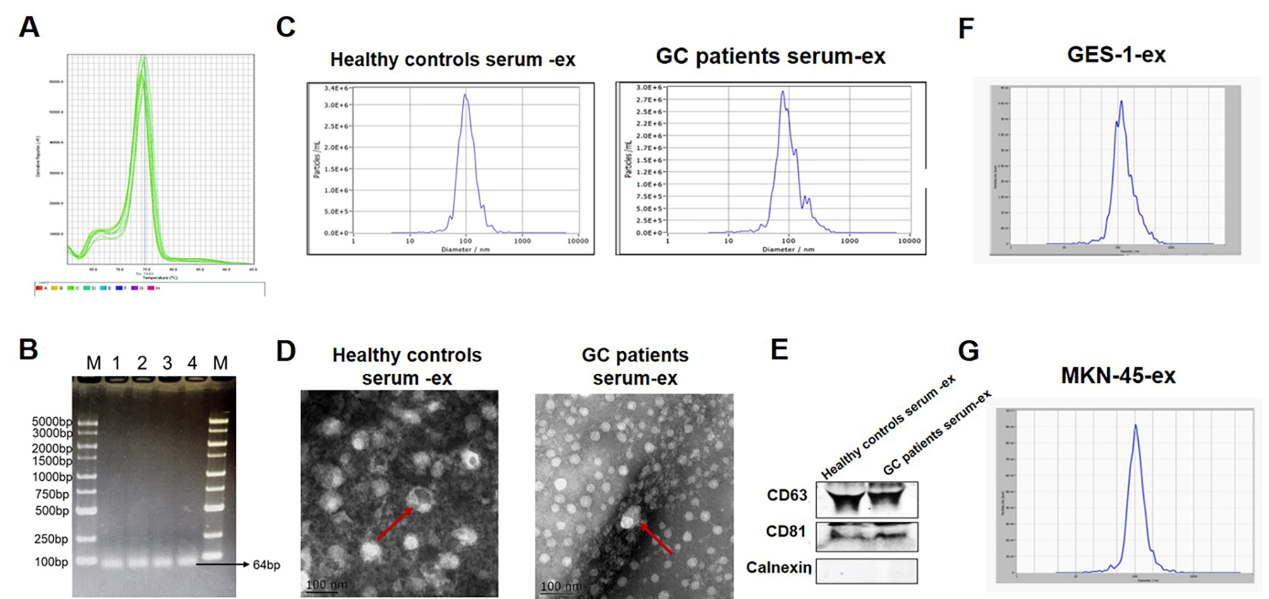


A. The dissolution curve for hsa_circ_000200. B. The results of agarose gel electrophoresis. C. Nanoparticle Tracking Analysis (NTA) detected the particle size of serum exosomes in healthy controls and GC patients. D. The morphology of serum exosomes in healthy controls and GC patients under transmission electron microscope. E. Protein identification of serum exosomes in healthy controls and GC patients. F. NTA detected the particle size of exosomes from GES-1 cells. G. NTA detected the particle size of exosomes from MKN-45 cells.

**Figure S2**


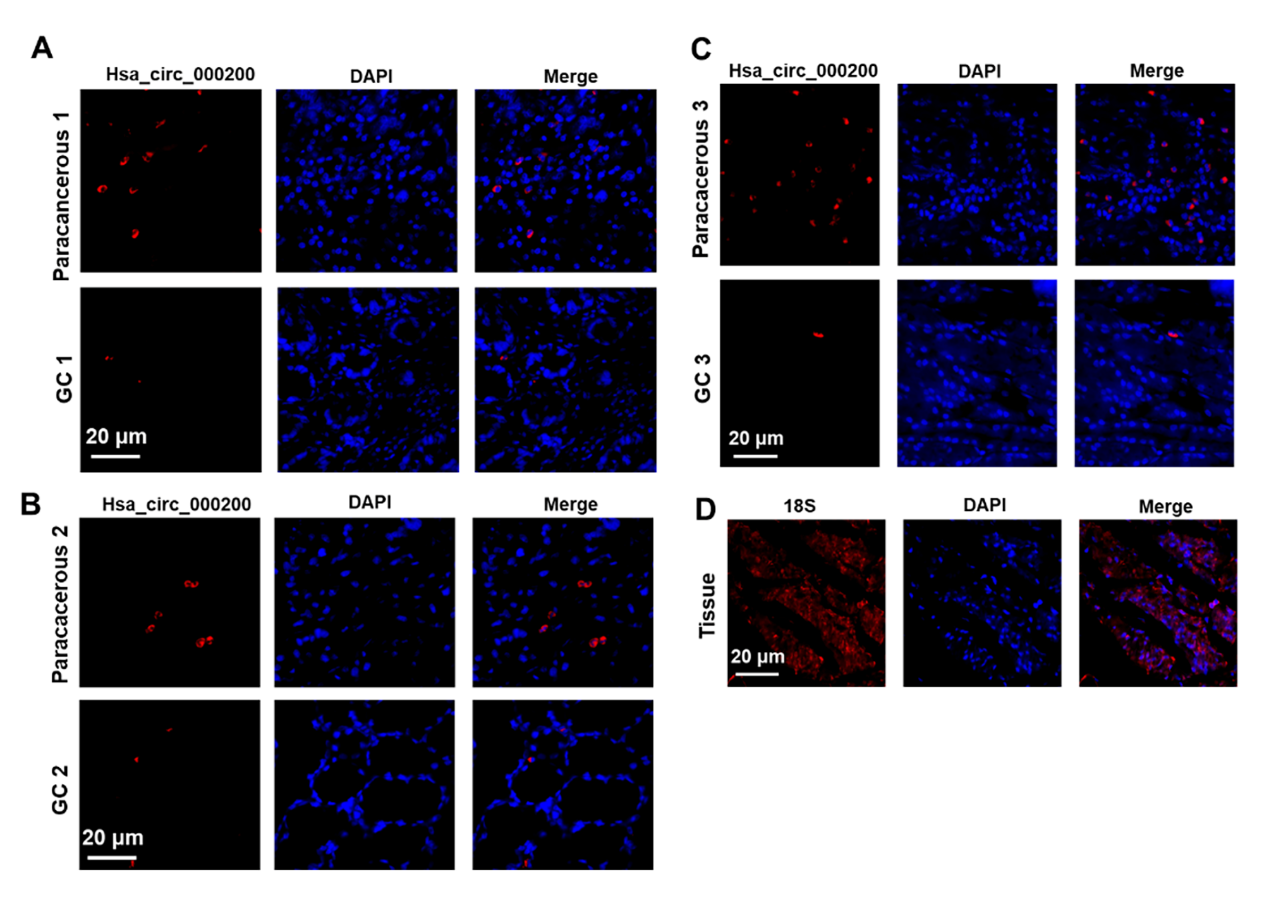


A/B/C. The localization of hsa_circ_000200 in GC and paired paracancer tissues. D. The localization of 18S in GC and paired paracancer tissues.

**Figure S3**


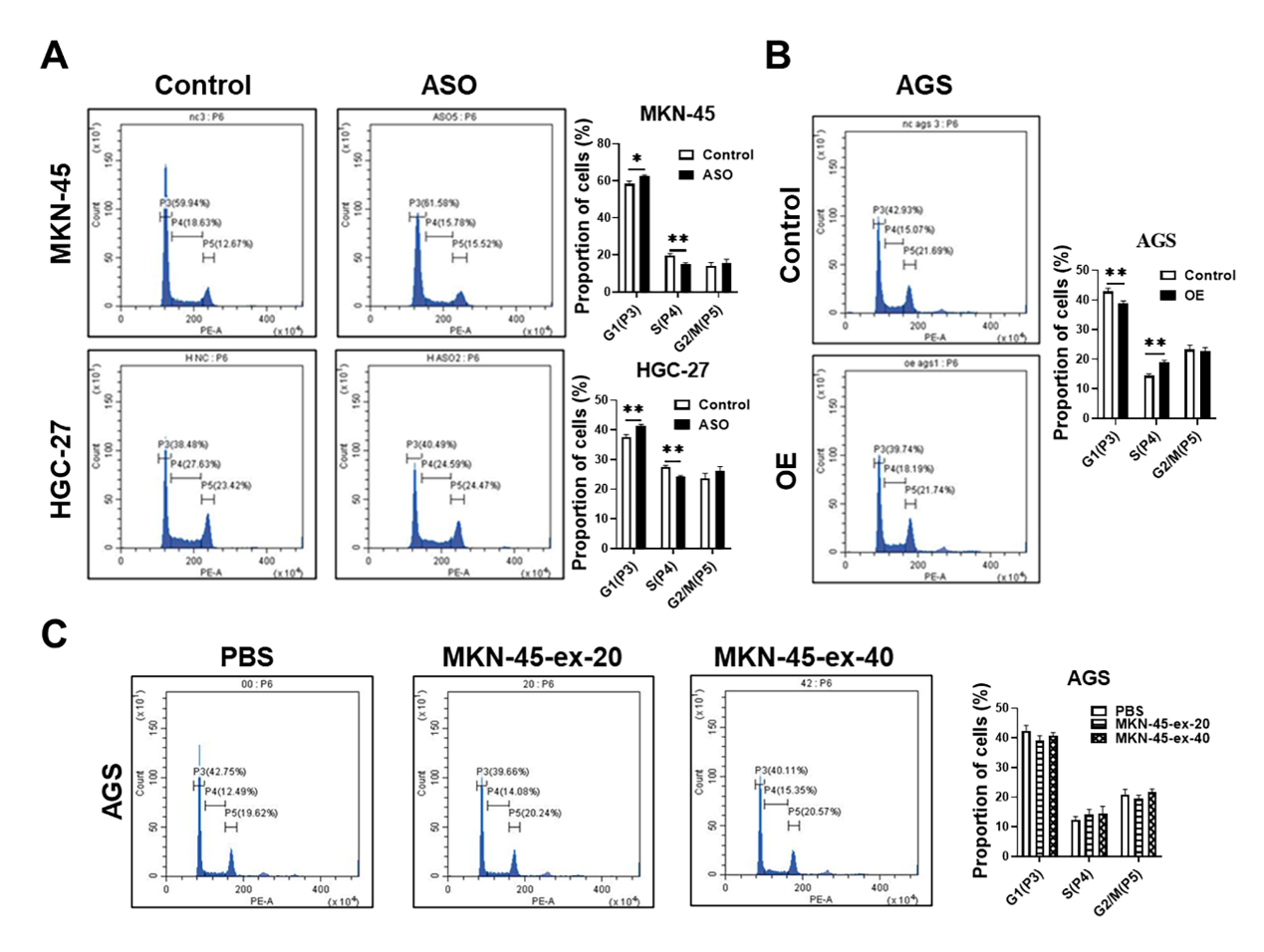


A. The results of cell cycle experiments after hsa_circ_000200 knockdown in MKN-45 and HGC-27 cells. B. The results of cell cycle experiments after hsa_circ_000200 overexpression in AGS cells. C. The results of cell cycle experiments after co-culture of exosomes.

**Figure S4**


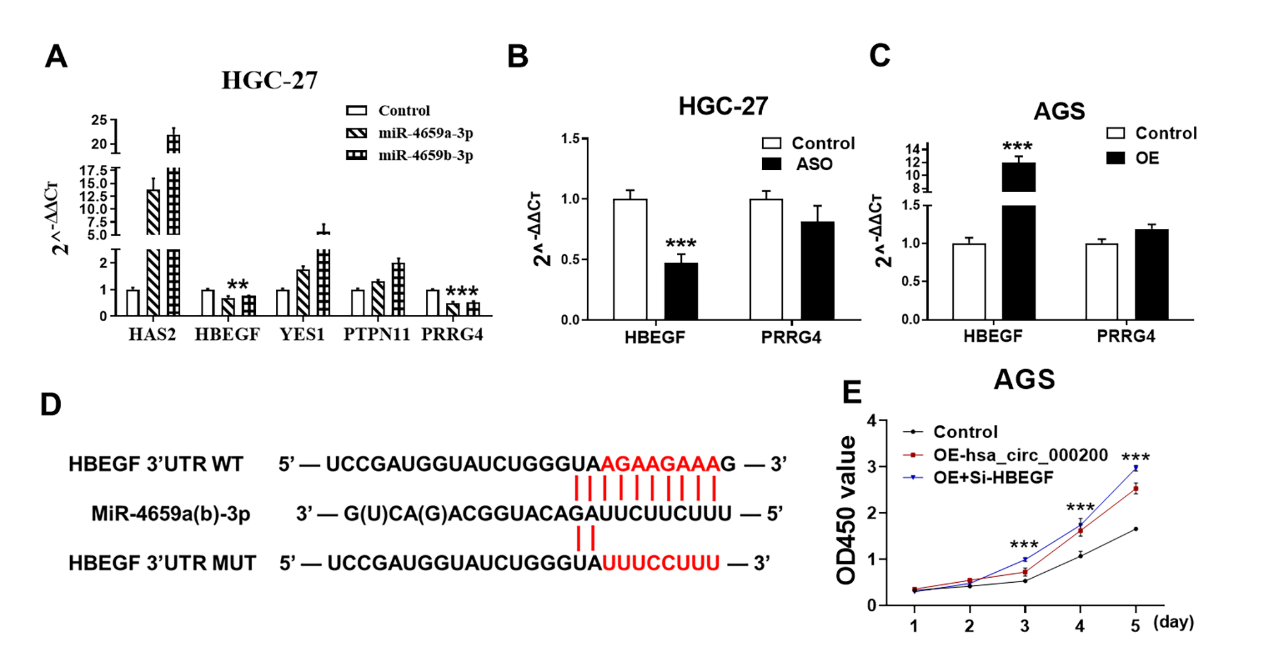


A. Validation of the levels of mRNA that may bind to miR-4659a/b-3p after miR-4659a/b-3p mimics transfection in HGC-27 cells. B. Validation of the levels of HBEGF and PRRG4 after hsa_circ_000200 knockdown in HGC-27 cells. C. Validation of the levels of HBEGF and PRRG4 after hsa_circ_000200 overexpression in AGS cells. D. The potential binding site of HBEGF in miR-4659a/b-3p was predicted by bioinformatic software. E. Assessment of the proliferation in AGS cells transfected with hsa_circ_000200 plasmid and si-HBEGF by CCK8 assay.

**Figure S5:**

**
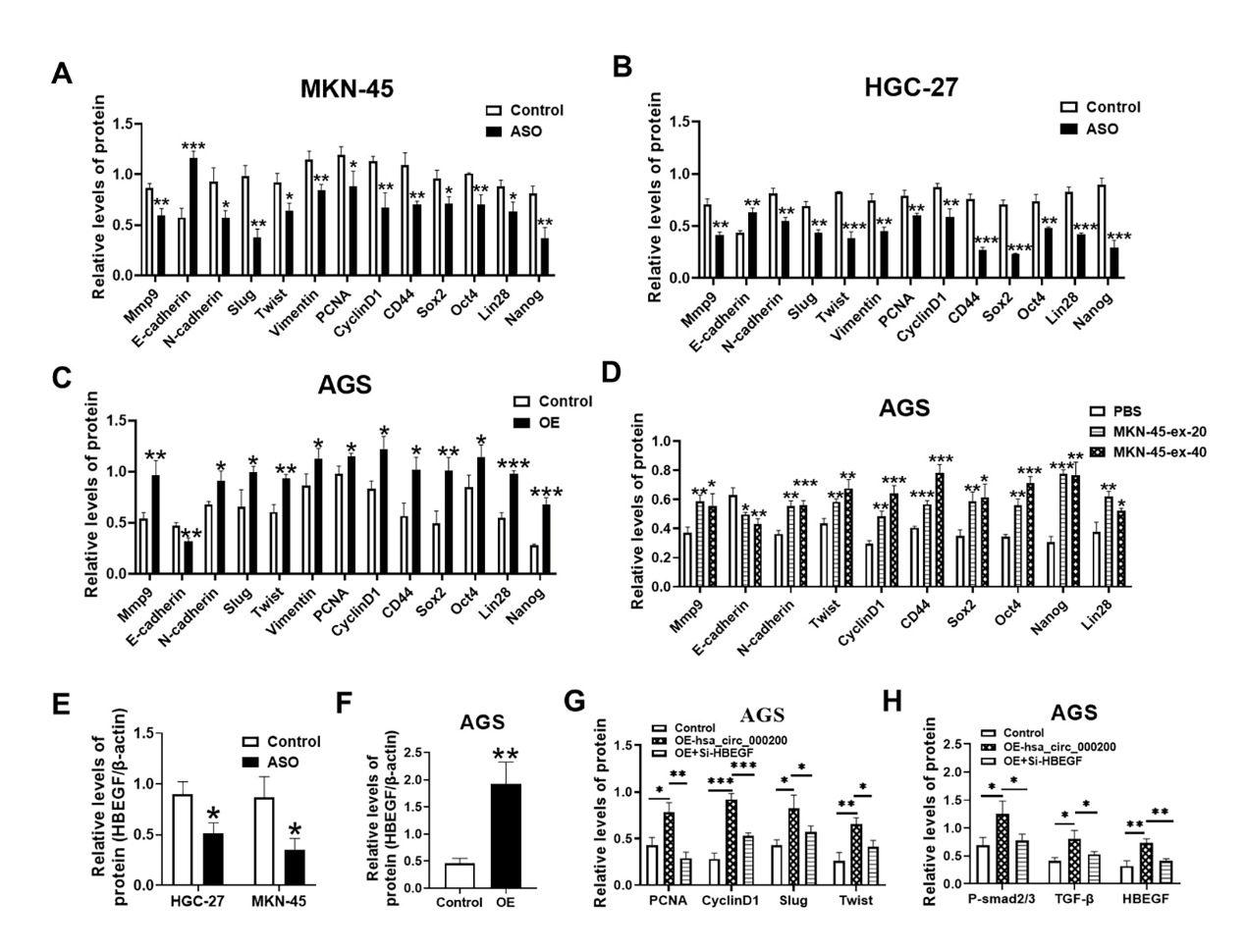
**

A-H. Relative levels of protein. **P* < 0.05; ***P* < 0.01; ****P* < 0.001.
